# Supplementary material for: Integrating Bioinformatics and Experimental Validation Identifies SCD as a Ferroptosis-Related Immune Regulator and Therapeutic Target in Crohn’s Disease
Source: Int J Mol Sci. 2025 Dec 19;27(1):19. doi: 10.3390/ijms27010019 (PMC12786048; doi:10.3390/ijms27010019)
Supplement: Supplementary file 1 [file ijms-27-00019-s001.zip › ijms-3926966-supplementary.pdf]

International Journal of
Molecular Sciences

MDPI

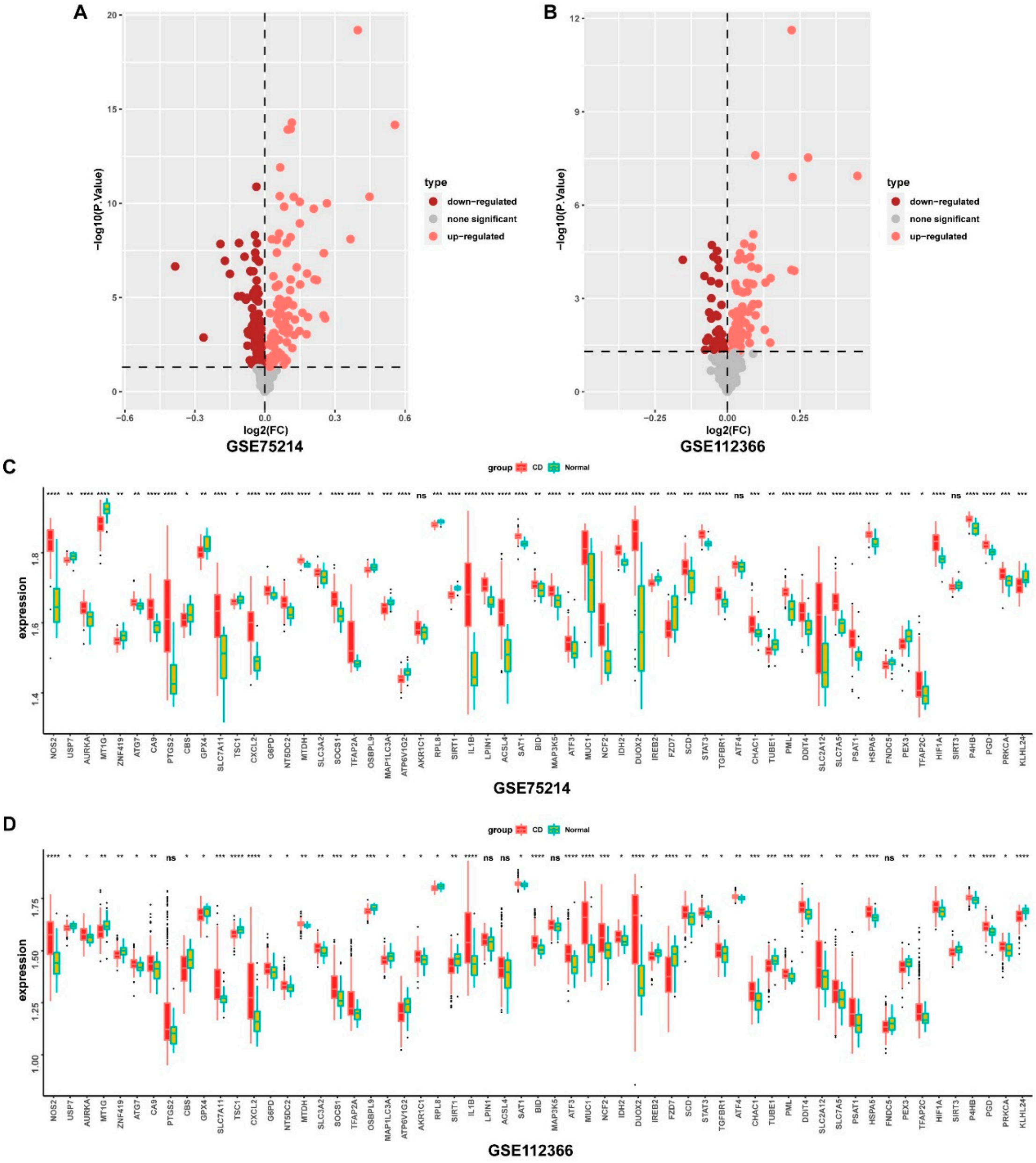

Supplementary Figure S1. Differential gene expression analysis in Crohn's disease (CD) patients compared to healthy controls using microarray datasets GSE75214 and GSE112366. Volcano plot for the GSE75214 dataset(A) and for the GSE112366 dataset(B). Box-plot displaying expression levels of selected genes in the GSE75214 dataset(C) and for the GSE112366 dataset(D).

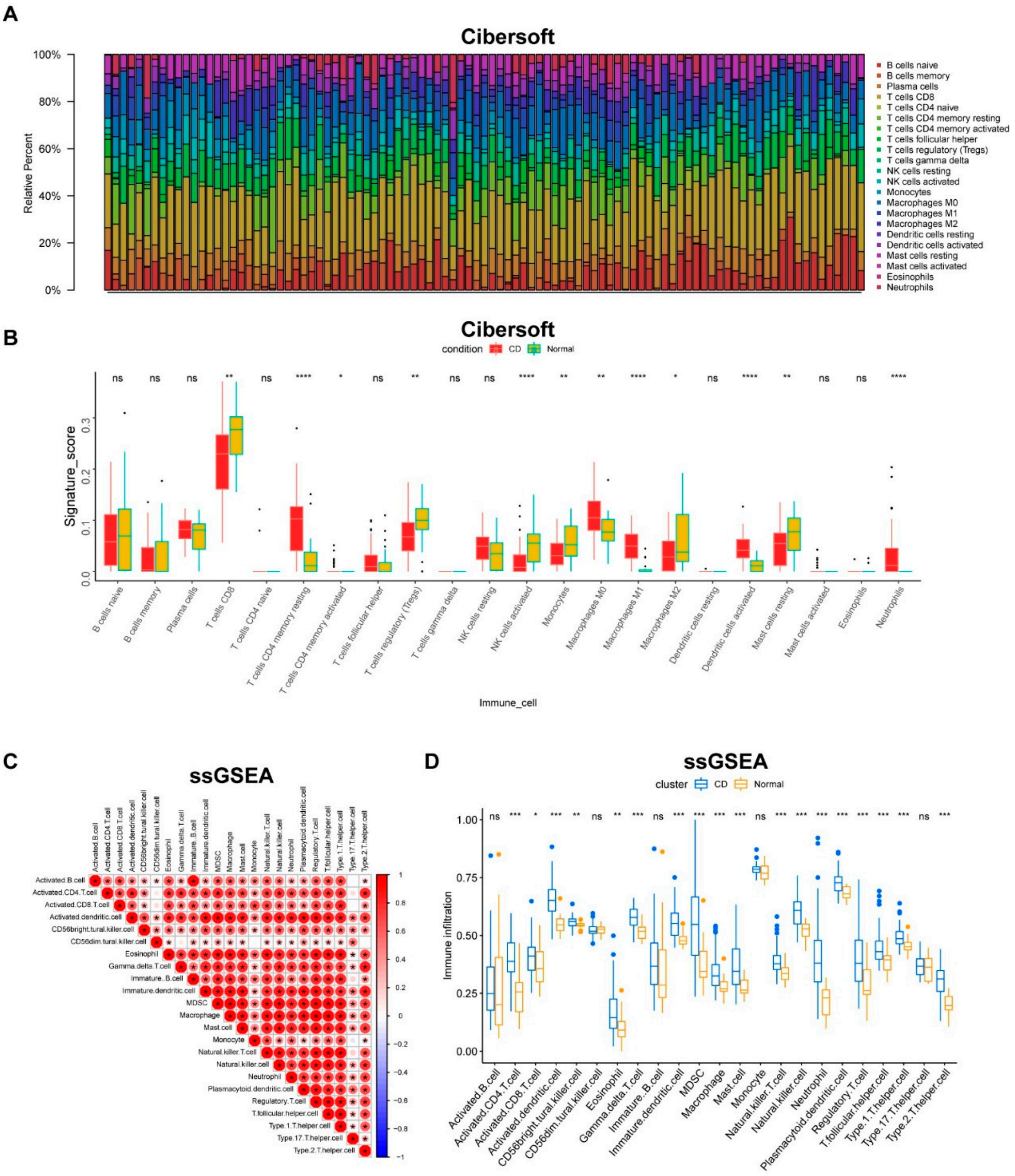

Supplementary Figure S2. Comprehensive analysis of immune cell infiltration in the microenvironment of Crohn's disease (CD). Analysis based on the Cibersort algorithm. **(A)** Stacked bar plot showing the relative proportions of various immune cell subsets across individual samples. **(B)** Box plots comparing the signature scores of different immune cell types between the CD and Normal groups. **(C, D)** Analysis based on the ssGSEA algorithm. **(C)** Heatmap displaying the enrichment scores of immune cell populations across samples or groups. **(D)** Box plots comparing the immune infiltration levels of various cell types between the CD and Normal groups.

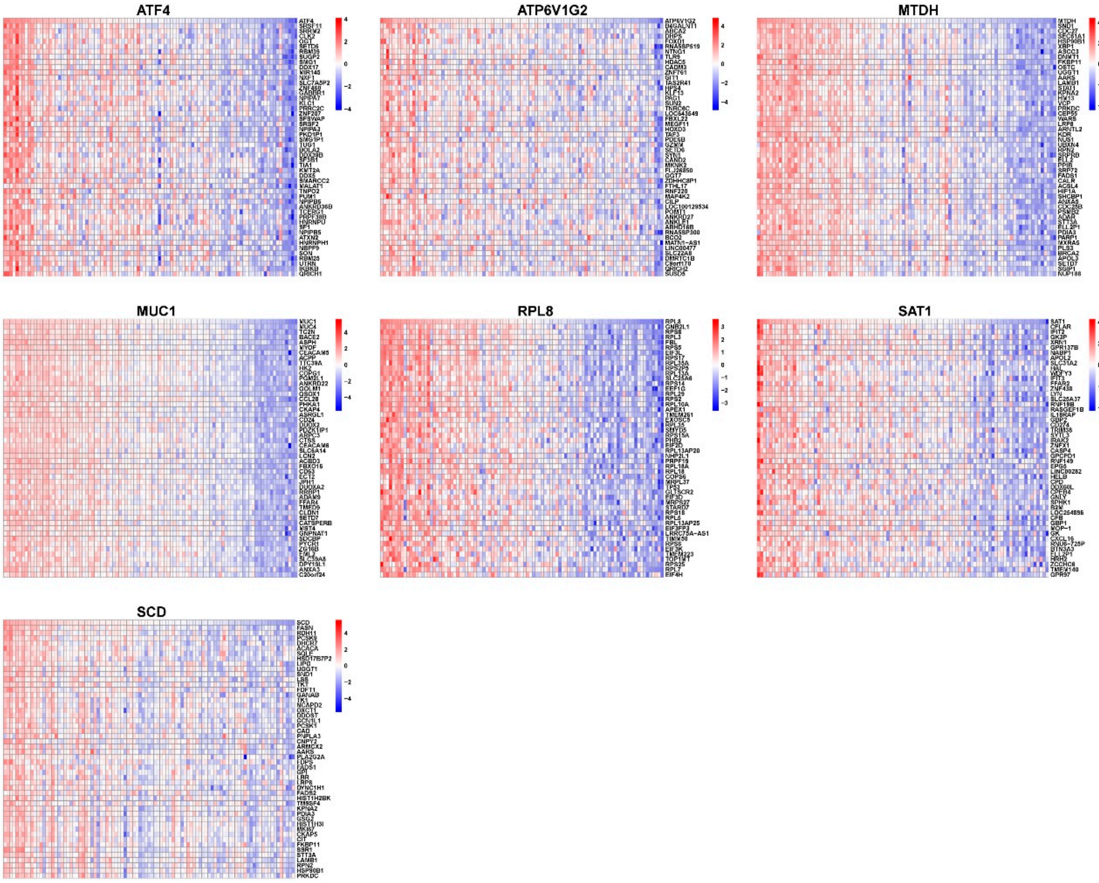

Supplementary Figure S3. Analysis of hub FEDG expression patterns in Crohn's disease. The heatmaps depict the normalized expression profiles of seven hub FEDGs(ATF4, ATP6V1G2, MTDH, MUC1, RPL8, SAT1, and SCD) across individual samples from the GSE75214 dataset. (TOP 50 gene)

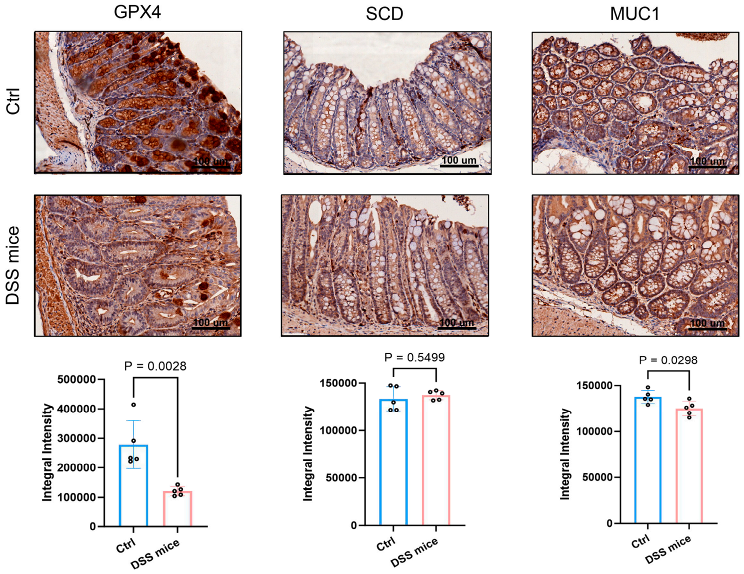

Supplementary Figure S4. Immunohistochemical (IHC) validation of hub ferroptosis-related differentially expressed genes (FEDGs) in a DSS-induced murine model of Crohn's disease. Representative immunohistochemical images of GPX4, SCD, and MUC1 in colon tissues from the health control and DSS-treated mice (scale bar: 100 µm), with quantitative analysis of protein levels shown as Integrated Density (IntDen). Data are presented as mean ± SD.

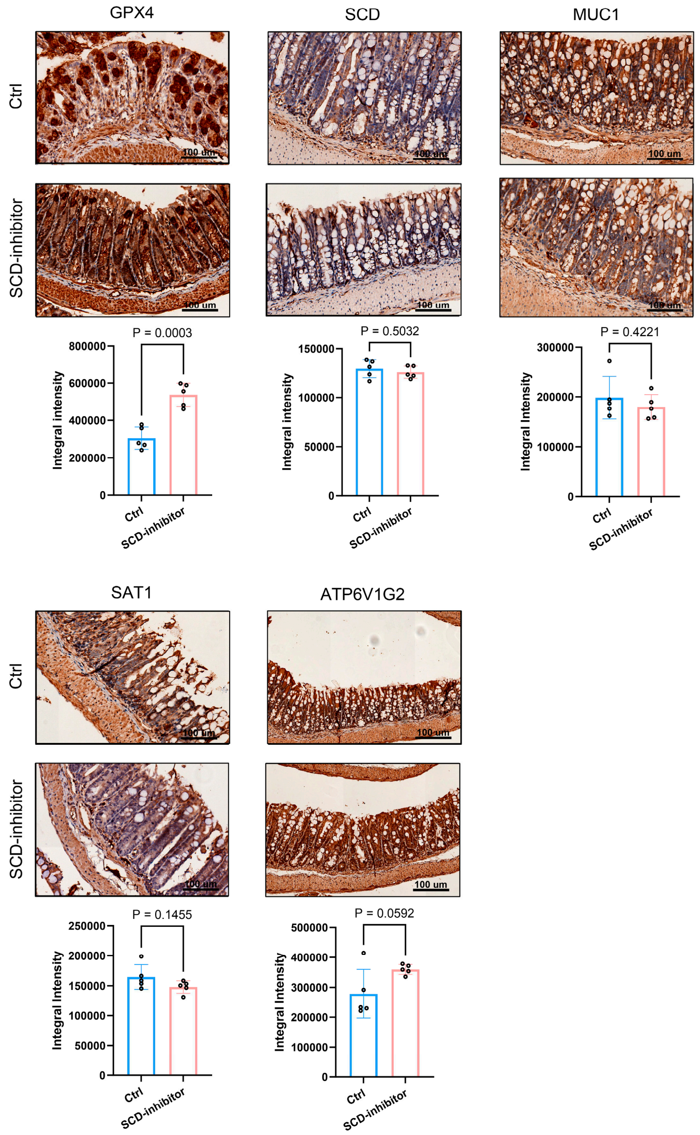

Supplementary Figure S5. Protein expression of hub ferroptosis-related genes in the DSS-induced colitis model upon SCD inhibition. Representative immunohistochemical images of GPX4, SCD, MUC1, SAT1 and ATP6V1G2 in colon tissues from the DSS-control and SCD-inhibitor-treated groups (scale bar: 100 µm), with quantitative analysis of protein levels shown as Integrated Density (IntDen). Data are presented as mean ± SD.

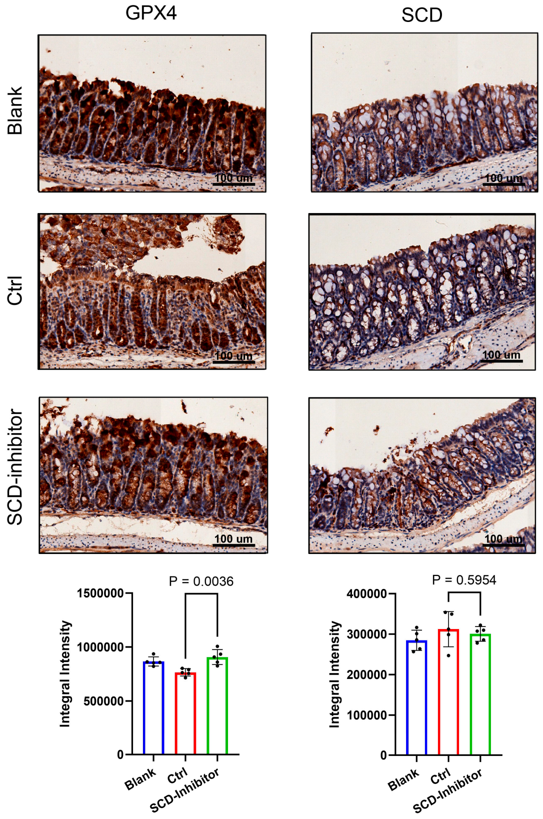

Supplementary Figure S6. Protein expression of GPX4 and SCD in the TNBS-induced colitis model upon SCD inhibition. Representative immunohistochemical images of GPX4 and SCD in colon tissues from the Blank, Ctrl, and SCD-inhibitor groups from TNBS model (scale bar: 100 μm), with quantitative analysis of protein levels shown as Integrated Density (IntDen). Data are presented as mean ± SD.

Supplementary Table S1. Primer of real-time PCR

Gene Symbol	Primer	
Tnf	Forward Primer	CCCTCACACTCAGATCATCTTCT
	Reverse Primer	GCTACGACGTGGGCTACAG
Ifng	Forward Primer	ATGAACGCTACACACTGCATC
	Reverse Primer	CCATCCTTTTGCCAGTTCCTC
Il1b	Forward Primer	GCAACTGTTCCTGAACTCAACT
	Reverse Primer	ATCTTTTGGGGTCCGTCAACT
Il6	Forward Primer	TAGTCCTTCCTACCCCAATTTCC
	Reverse Primer	TTGGTCCTTAGCCACTCCTTC
Il17a	Forward Primer	TTTAACTCCCTTGGCGCAAAA
	Reverse Primer	CTTTCCCTCCGCATTGACAC
Il10	Forward Primer	GCTCTTACTGACTGGCATGAG
	Reverse Primer	CGCAGCTCTAGGAGCATGTG

Cxcl1	Forward Primer	CTGGGATTCACCTCAAGAACATC
	Reverse Primer	CAGGGTCAAGGCAAGCCTC
Cxcl2	Forward Primer	CCAACCACCAGGCTACAGG
	Reverse Primer	GCGTCACACTCAAGCTCTG
Cxcl3	Forward Primer	TGAGACCATCCAGAGCTTGACG
	Reverse Primer	CCTTGGGGGTTGAGGCAAACTT
Cxcl5	Forward Primer	CCGCTGGCATTTCTGTTGCTGT
	Reverse Primer	CAGGGATCACCTCCAAATTAGCG
Cxcl10	Forward Primer	ATCATCCCTGCGAGCCTATCCT
	Reverse Primer	GACCTTTTTTGGCTAAACGCTTTC
